# Rationally Modified SARS-CoV-2 Spike Protein Impairs ACE2 Binding While Preserving Immunogenicity in Mice

**DOI:** 10.3390/vaccines14070568

**Published:** 2026-06-27

**Authors:** Elia Tamagnini, Luca Simonelli, Martin Palus, Tanja Rezzonico Jost, Edoardo Lazzarini, Davide Mangani, Václav Hönig, Markéta Dvořáková, Dominik Arbon, Federica Gambini, Sara Lestani, Fabio Grassi, Lucio Barile, Mattia Pedotti, Radislav Sedlacek, Luca Varani

**Affiliations:** 1 Institute for Research in Biomedicine, Università della Svizzera italiana (USI), 6500 Bellinzona, Switzerland; elia.tamagnini@irb.usi.ch (E.T.); luca.simonelli@irb.usi.ch (L.S.); davide.mangani@irb.usi.ch (D.M.); sara.lestani@irb.usi.ch (S.L.); mattia.pedotti@irb.usi.ch (M.P.); 2 Laboratory of Arbovirology, Institute of Parasitology, Biology Centre of the Czech Academy of Sciences, CZ-37005 České Budějovice, Czech Republic; palus@paru.cas.cz (M.P.); honig@paru.cas.cz (V.H.); marketa.spevakova@paru.cas.cz (M.D.); 3 Laboratory of Emerging Viral Infections, Veterinary Research Institute, CZ-62100 Brno, Czech Republic; 4 Department of Experimental Biology, Faculty of Science, Masaryk University, CZ-62500 Brno, Czech Republic; 5 Institute of Oncology Research, 6500 Bellinzona, Switzerland; tanja.rezzonico@ior.usi.ch; 6 Institute for Translational Research (IRT), Faculty of Biomedical Sciences, Università Svizzera Italiana (USI), Ente Ospedaliero Cantonale (EOC), 6500 Bellinzona, Switzerland; edoardo.lazzarini@eoc.ch (E.L.); lucio.barile@usi.ch (L.B.); 7 Cardiovascular Theranostics Group, Istituto Cardiocentro Ticino, Ente Ospedaliero Cantonale (EOC), 6900 Lugano, Switzerland; 8 Euler Institute, Faculty of Biomedical Sciences, Università della Svizzera Italiana (USI), 6900 Lugano, Switzerland; 9 Czech Centre for Phenogenomics, Institute of Molecular Genetics of the Czech Academy of Sciences, 252 50 Vestec, Czech Republic; dominik.arbon@img.cas.cz (D.A.); radislav.sedlacek@img.cas.cz (R.S.); 10 Laboratory of Transgenic Models of Diseases, Institute of Molecular Genetics of the Czech Academy of Sciences, 142 20 Prague, Czech Republic; federica.gambini@img.cas.cz; 11 National Institute for Molecular Genetics “Romeo ed Enrica Invernizzi” INGM, 20122 Milan, Italy; grassi@ingm.org; 12 Department of Medical Biotechnology and Translational Medicine, University of Milan, 20129 Milan, Italy

**Keywords:** vaccine design, protein engineering, off-target interference, SARS-CoV-2, spike protein, ACE2 receptor

## Abstract

Background: While vaccines are designed to elicit targeted immune responses, in some cases, the immunogenic molecules employed can inherently interact with broader host cellular pathways as a secondary consequence. This phenomenon can be exemplified by COVID-19 vaccines. COVID-19 vaccines, including mRNA platforms, use the SARS-CoV-2 spike protein as an immunogen to induce the production of neutralizing antibodies. The spike protein binds the ACE2 (angiotensin-converting enzyme 2) receptor on human cells, mediating viral entry and infection. ACE2 is widely expressed across multiple tissues and is a key component of the renin–angiotensin–aldosterone system (RAAS) that acts as a homeostatic regulator of systemic and local blood flow, blood pressure, cardiac function, fluid balance and immunity. Some studies have proposed the interaction between the spike protein and ACE2 as a possible contributing factor to rare adverse effects observed following COVID-19 vaccination, including myocarditis, pericarditis, thrombosis, and reported alterations in blood pressure, though these mechanisms remain to be fully elucidated. Objectives: As a proof-of-concept approach in vaccine antigen development, we engineered SARS-CoV-2 spike mutants with impaired binding to the host receptor ACE2. Methods: By rational design, we produced and validated in vitro and in vivo spike point mutants that do not effectively bind ACE2. Results: The engineered spike mutants do not effectively bind the human entry receptor ACE2 while retaining the immunogenic properties equal to or better than the wild type spike and thus generate a protective response in animals when used as a vaccination agent. Conclusions: By establishing a straightforward molecular strategy for rational vaccine design, this work demonstrates the feasibility of limiting specific antigen–host receptor interactions while maintaining immunogenicity. This approach may be applicable to future vaccination strategies where antigen interaction with host cells could potentially interfere with physiological pathways.

## 1. Introduction

During the COVID-19 pandemic, a global vaccination campaign resulted in the administration of over thirteen billion doses [1,2]. While vaccines have proven safe and effective, like any therapeutic intervention, they can cause adverse effects (AEs). Whereas most reported AEs are mild and temporary, including fever, fatigue, headache, chills, myalgia, arthralgia and lymphadenopathy [3,4], some more severe but less frequent AEs have been described. These include anaphylaxis [5,6,7], autoimmune reactions [8], cardiovascular events (e.g., myocarditis, pericarditis) [9,10,11], thrombosis [12,13], blood pressure alterations [14,15], and menstrual disturbances in young girls after COVID-19 vaccination [16]. Cardiovascular events were particularly notable in younger individuals [17], leading some countries to discourage specific vaccines for males under 30 [18]. Although the exact number of cases varies vastly across scientific literature, there is consensus on the incidence of myocarditis in young males, up to 40 cases per 100,000 individuals under 40, with the highest risk among the 12–24 age group [14]. The total incidence of total venous thrombosis after vaccination is estimated at 28 per 100,000, with a predominance of women (70%) of whom about half are under the age of 50 [19,20,21].

The molecular mechanisms underlying these cardiovascular AEs remain to be determined. One hypothesis that has been proposed implicates interaction between the SARS-CoV-2 spike protein and its human entry receptor, angiotensin-converting enzyme (ACE2) [22,23]. ACE2 is widely expressed in human tissues, particularly in the cardiovascular system where it regulates the renin–angiotensin–aldosterone system (RAAS) [24,25,26]. RAAS plays a crucial role in cardiovascular homeostasis, and it has been hypothesized that this system could potentially provide a link between vaccination and some observed AEs. Some research has suggested that spike protein binding to ACE2 might disrupt RAAS balance, though the clinical relevance of these findings remains to be established [24,27].

We hypothesized that preserving the ability of the spike vaccination agent to generate a protective immune response while impairing its interaction with ACE2 could provide a valuable research tool for investigating the potential role of spike-ACE2 interactions in vaccination outcomes. We set forth to generate spike protein mutants with compromised ACE2 binding affinity. We show that these mutants retain the ability to generate a protective response, which was equal to or better than the wild-type spike.

Altogether, our findings demonstrate the technical feasibility of impairing specific antigen–receptor interactions while maintaining immunogenicity. This rational design approach may inform future vaccine development strategies where antigen interactions with host receptors could potentially interfere with physiologically relevant pathways beyond COVID-19.

## 2. Materials and Methods

### 2.1. Spike-ACE2 Structural Analysis

PyMOL [28] was employed to visualize the 3D structure of the spike-ACE2 complex (PDB code: 7XO5) [29], defining the SARS-CoV-2 RBD interaction interface as any residue located within 7 Å of ACE2. In this study, the visual structural analysis was utilized to map critical chemical interactions and characterize the essential residues driving the RBD-ACE2 binding affinity.

### 2.2. Protein Expression and Purification

Recombinant RBD mutants: to express SARS-CoV-2 RBD, a codon-optimized gene encoding residues 316–534 (GenBank (https://www.ncbi.nlm.nih.gov/genbank/): UFO69279.1 (accessed on 1 June 2023)) with a C-terminal 8× HisTag was synthesized and cloned into the mammalian expression vector pcDNA3.1(+) by Genscript. RBD mutations were introduced in the gene by single-point mutations (Genscript, Piscataway, NJ, USA). The resulting variants were produced by transient PEI transfection in Expi293F cells (ThermoFisher, Waltham, MA, USA). Six days post-transfection, the proteins were purified from the cell supernatants using affinity chromatography (HiTrap Chelating HP, Cytiva, Marlborough, MA, USA), followed by size-exclusion chromatography (SEC 75, Cytiva).

Recombinant ACE2: ACE2 fused to the Fc region of mouse IgG at the C terminus was synthesized and cloned into the mammalian expression vector pcDNA3.1(+). ACE2 was produced by transient PEI transfection in Expi293F cells (Thermo Fisher), purified from the cell supernatants 6 days after transfection by affinity chromatography (HiTrap Chelating HP, Cytiva) followed by size exclusion chromatography (SEC 75, Cytiva).

All proteins were analyzed by SDS–PAGE and underwent quality control and biophysical characterization to ensure functionality, stability, lack of aggregation and batch-to-batch reproducibility.

### 2.3. Analysis of RBD-ACE2 Binding Interactions

ELISA: 96-well ELISA plates were coated at 4 °C overnight with 180 nM RBD, and then washed and blocked with PBS containing 10% FBS. ACE2 was then added in a 1:3 serial dilution, starting from 250 nM for wtRBD and R493A and 1000 nM for the remaining mutants. After a 1 h incubation at room temperature and subsequent washing, the binding between the RBD mutants and ACE2 was detected using goat anti-mouse IgG conjugated to alkaline phosphatase (1:500 dilution; Southern Biotech, Birmingham, AL, USA).

The experiment was performed in duplicate, and the mean of the two replicates was reported; error bars represent the standard deviation of the measured values. Absorbance was measured using a microplate reader with Gen5 software version 1.11.5 (BioTek Instruments, Winooski, VT, USA).

Surface Plasmon Resonance (SPR): RBD mutants′ binding kinetics were characterized at 25 °C using a Biacore 8K instrument (GE Healthcare, Chicago, IL, USA). The running buffer consisted of 10 mM HEPES (pH 7.4), 150 mM NaCl, 3 mM EDTA, and 0.005% (*v*/*v*) Tween-20. RBD protein was covalently immobilized at 150 nM onto CM5 sensor chips (Cytiva, Marlborough, MA, USA) via standard amine coupling chemistry. Single-cycle kinetics were performed by sequentially injecting five increasing concentrations of recombinant ACE2, utilizing a two-fold serial dilution with a maximum concentration of 800 nM (50, 100, 200, 400, and 800 nM).

### 2.4. RBD Binding to HEK293 Cells Overexpressing ACE2

HEK293 cells stably transfected with ACE2-GFP were seeded at 20,000 cells per well in 100 µL of medium using a flat-bottom 96-well plate, then incubated for two days. A 1:2 serial dilution of the RBD mutants, starting at 1000 nM, was added and incubated for 1 h. The cells were then washed with PBS containing 1% FBS, followed by the addition of 50 µL of Alexa Fluor^®^ 647-conjugated AffiniPure Rabbit Anti-His Tag (diluted 1:4000) for 30 min. After two additional washes, 1 µL of 7-AAD was added to each well immediately before analyzing the samples via flow cytometry (FACS).

### 2.5. Immunofluorescence Assay

Calu-3 human adenocarcinoma cell lines (ATCC) and Human Cardiac Microvascular Endothelial Cells (PromoCell) were cultured according to the suppliers’ recommendations. Once cells reached approximately 80% confluence, they were treated with either wtRBD or H505A mutant. Endothelial cells were exposed to 2000 nM RBD for 1 h at 37 °C, while Calu-3 cells were exposed to 200 nM RBD under the same conditions. Control cells (Ctr) were incubated with PBS under identical conditions to assess background autofluorescence and non-specific antibody binding. Cells were then fixed in methanol and subjected to immunostaining using anti-His Tag Alexa Fluor 647 (COD) and anti-ACE2 (Genetex, Irvine, CA, USA; GTX101395) antibodies. Confocal images were acquired using a Leica SP8 microscope and analyzed with Fiji (ImageJ 1.54e; Java 1.8.0_322; National Institutes of Health, Bethesda, MD, USA). Briefly, cells were segmented based on ACE2 expression patterns, and His Tag fluorescence intensity was quantified for each individual cell. A minimum of 40 cells per experimental group was analyzed.

### 2.6. Mice Immunization Experiment

Four 6-week-old female BALB/c mice were vaccinated for each RBD mutant and wtRBD. The mice were housed in individually ventilated cages (Techniplast) in a specific pathogen-free (SPF) facility with sterilized wood-chip bedding, under standardized conditions (20° ± 2 °C, 55 ± 8% relative humidity, and 12 h/12 h light/dark cycle). A diet of sterilized pellets and water was provided ad libitum. All animal experiments were performed in accordance with the Swiss Federal Veterinary Office guidelines and approved by the Ethical Committee of the Cantonal Veterinary Office, with authorization number 35619. A 1:1 mixture (100 μL total) of 0.5 mg Imject Alum adjuvant (Thermo Scientific, Waltham, MA, USA; 10475325) and RBD (10 μg, 7.2 μM) was administered subcutaneously to each mouse. Prior to injection, the solution was mixed for at least 30 min to ensure effective antigen adsorption. Three doses were administered every 2 weeks, and the blood was taken 20 days after the last shot.

### 2.7. Determination of Serum Antibody Titers in Vaccinated Mice

The 96-well ELISA plates were coated overnight at 4 °C with 180 nM wtRBD, and afterwards washed and blocked with PBS containing 10% FBS. Sera were initially diluted 1:3, followed by three-fold serial dilutions. After a 1 h incubation at room temperature and subsequent washing, the binding between serum antibodies and wtRBD was detected using goat anti-mouse IgG conjugated to alkaline phosphatase (1:500 dilution; Southern Biotech, Birmingham, AL, USA). The experiment was performed in duplicate, and the mean of the two replicates was reported; error bars represent the standard deviation of the measured values. Absorbance was measured using a microplate reader with Gen5 software version 1.11.5 (BioTek Instruments, Winooski, VT, USA).

### 2.8. Inhibition of ACE2–RBD Interaction by Mice Sera

The 96-well ELISA plates were coated overnight at 4 °C with 100 nM ACE2, and afterwards washed and blocked with PBS containing 10% FBS. Sera were serially diluted 1:2 and then incubated with 20 nM wtRBD for 15 min at room temperature. Complexes were added to immobilized ACE2; after a 1 h incubation at room temperature and subsequent washing, the binding between ACE2 and wtRBD was detected using goat anti-His tag IgG conjugated to horse radish peroxidase (dilution 1:10,000). Absorbance was measured using a microplate reader with Gen5 software version 1.11.5 (BioTek Instruments, Winooski, VT, USA).

### 2.9. Plaque Reduction Neutralization Test

The plaque reduction neutralization test (PRNT) was performed using the live SARS-CoV-2 Omicron variant (B.1.1.529 17577/21). The PRNT served to determine the neutralization capacity of sera from mice immunized with RBD mutants. Sera from mice immunized with the H505A mutant (mouse no. 23), wtRBD (mouse no. 27), and a PBS/Imject Alum control (mouse no. 1) were tested in duplicate. Serial dilutions of the sera were incubated with 50 plaque-forming units (PFUs) of SARS-CoV-2 at 37 °C for 2 h. The serum-virus mixtures were then added to Vero E6 cells (ATCC CRL-1586) monolayers in 24-well plates and incubated at 37 °C for an additional hour. Following incubation, cells were overlaid with 1.5% (*w*/*v*) carboxymethylcellulose in Dulbecco’s Modified Eagle Medium (DMEM Low Glucose with Stable Glutamine and Sodium Pyruvate; antibiotics/antimycotics; Biosera, Cholet, France) supplemented with 5% FBS. Plates were incubated at 37 °C for 4 days. After incubation, cells were washed in PBS, and SARS-CoV-2 plaques were visualized by staining with naphthalene black for 45 min at room temperature. Following a final water wash, plaques were quantified. The antibody neutralization titer was defined as the reciprocal of the highest dilution resulting in a 50% reduction in infection (PRNT50). Data were fitted to a 4-parameter logistic (4PL) sigmoidal dose–response curve using GraphPad Prism (GraphPad Software Inc., San Diego, CA, USA; software version 10.6.1 (892)). All experiments were conducted in a Biosafety Level 3 containment laboratory.

## 3. Results

With the aim of generating a spike vaccination agent that cannot interact with ACE2, we first identified the spike residues that are critical for such interaction. Visual structural analysis of the spike-ACE2 complex structure [29] suggests a set of residues whose mutation results in impaired binding energy.

### 3.1. The RBD Mutants Have Reduced Binding Affinity for Recombinant ACE2

**Figure 1:**
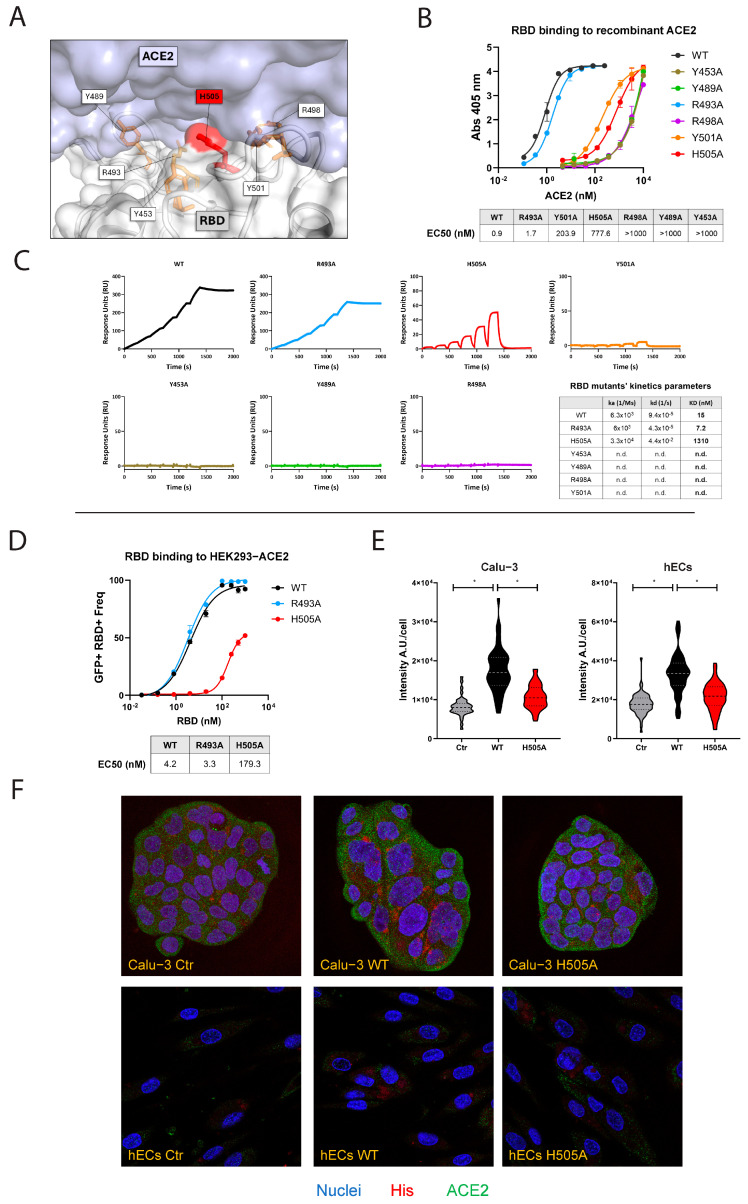
Spike point mutations affect ACE2 binding. (**A**) Cartoon representation of the RBD (grey)—ACE2 (blue) interface; pdb code 7XO5. Spike residues important for the interaction are in red and orange. (**B**) In vitro ELISA binding of wtRBD and mutants to recombinant ACE2 and their relative EC_50_. (**C**) SPR sensorgrams for wtRBD and mutants binding to recombinant ACE2, and their corresponding Kinetic Parameters. (**D**) In cell binding of wtRBD and H505A to HEK293 overexpressing ACE2, measured by FACS and their relative EC_50_. (**E**) Fluorescence intensity detected after binding of wtRBD and H505A on Calu-3 treated (**left**) and endothelial cells (**right**), physiologically expressing ACE2; measured by confocal microscopy. One-way ANOVA followed by Tukey′s post hoc test was used for multiple comparisons. Data are presented as violin plot s. * *p* value < 0.05 (**F**) Confocal microscopy images of Calu-3 and endothelial cells treated with wtRBD, H505A mutant, or PBS as a negative control (Ctr) to evaluate background fluorescence. Nuclei are stained in blue (DAPI), ACE2 in green, and RBD in red (detected via anti-His tag).

Spike RBD constructs with residues Y453, Y489, R493, R498, Y501 and H505 (Figure 1A) mutated to Alanine were produced in Expi293 cells and purified. We then assessed the ability of these mutants to bind ACE2 by ELISA (Figure 1B) and SPR (Figure 1C); five out of six displayed a significantly reduced binding affinity compared to the wild-type construct. R493A had binding affinity comparable to the wild-type RBD (wtRBD). Y501A was at least 200-fold weaker; H505A had ~5 times slower association and ~500-fold faster dissociation in SPR, and ~800 times weaker EC_50_ ELISA; R498A, Y489A and Y543A were several thousand-fold weaker.

### 3.2. The H505A Mutant Has Reduced Binding to ACE2 Expressing Cells

We showed that the RBD mutants have largely decreased binding to recombinant ACE2 in vitro. To further substantiate our approach, we investigated whether reduced ACE2 binding held true also when employing live-cell systems, first on cells engineered to express ACE2 on their surface and then on ACE2 naturally expressing cells.

**Figure 2:**
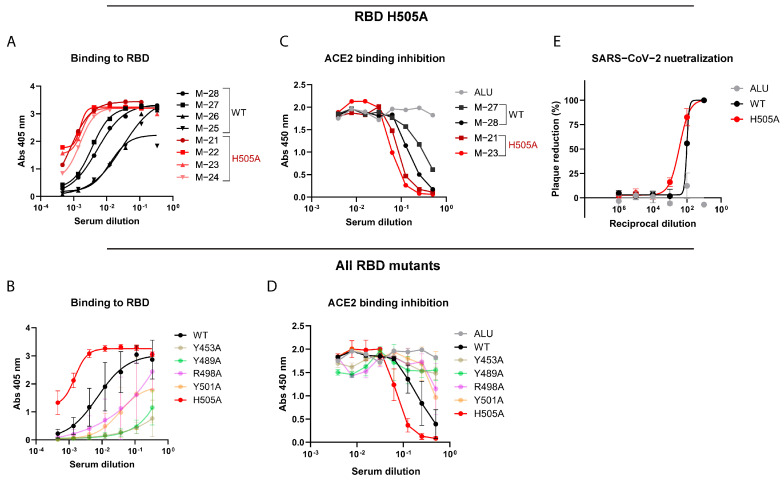
Immune response elicited by the RBD H505A mutant in mice. (**A**) ELISA evaluation of individual mouse sera (from mice immunized with wtRBD or H505A) binding to recombinant wtRBD; “M” indicates individual mouse numbers. (**B**) Average binding of sera for all RBD mutants to recombinant wtRBD via ELISA (*n* = 4 mice per group). (**C**) Inhibition of the wtRBD–ACE2 interaction by immune sera from mice immunized with wtRBD or H505A. (**D**) Average inhibition of wtRBD–ACE2 binding via ELISA (*n* = 4 mice per group) for all RBD mutants. (**E**) Neutralization of SARS-CoV-2 Omicron variant determined by plaque reduction assays.

The H505A mutant was selected due to its low affinity to ACE2 (Figure 1B,C) and robust immunogenicity in mice (Figure 2A,B); it also has a good production yield comparable to the wtRBD (~23mg/L in Expi293F) (Table S1). FACS confirmed that the H505A mutant exhibited significantly reduced interaction with HEK293 cells overexpressing ACE2-GFP in comparison to the wtRBD (Figure 1D). Lastly, we incubated naturally ACE2-expressing Calu-3 epithelial adenocarcinoma cells [30] and primary human endothelial cells [31] with wtRBD or H505A. Quantification of tag presence in target cells by confocal microscopy confirmed that H505A displayed less binding capacity to ACE2-expressing cells, compared to wtRBD (Figure 1E,F).

Altogether, our findings confirm that the H505A RBD mutant displays reduced interaction with the ACE2 receptor both in cell-free and cell-based assays in vitro.

### 3.3. Induction of wtRBD-Binding Antibodies by Engineered RBD Mutants

We immunized mice with each mutant with impaired binding to ACE2 to verify that they maintain satisfactory immunogenic properties resulting in a neutralizing response upon vaccination.

We employed the RBD as the immunogen, rather than the full-length spike protein, to ensure that the antibodies produced were specifically elicited by the RBD mutants, rather than by epitopes located in other regions of the spike protein that were not affected by mutations.

Different groups of mice were vaccinated with three doses administered every 2 weeks. Two weeks after the last dose, sera were collected and tested for their ability to effectively bind to recombinant, wtRBD. All mice treated with H505A generated antibodies binding to wtRBD at dilutions equal to or greater than those of sera from mice treated with wtRBD (Figure 2A,B). In contrast, wtRBD binding in sera from mice immunized with Y453A, Y489A, R498A, or Y501A was detectable only at high serum concentrations, indicating that these mutants failed to elicit an effective antibody response (Figure S1).

### 3.4. Sera from H505A-Immunized Mice Effectively Inhibit the RBD-ACE2 Interaction

Our findings confirm that mice generated antibodies against the entire RBD. However, we need to ensure that these antibodies are against the RBD-ACE2 interface, which is the main target for neutralizing antibodies. To address this, we tested the ability of the sera to inhibit the binding of wtRBD to ACE2 (Figure 2C,D). Sera from mice injected with Imject Alum alone were used as the negative control. Sera from mice immunized with H505A fully inhibited the binding between wtRBD and ACE2. No inhibition was observed in sera from mice immunized with the other mutants (Figure S2). Altogether, the designed RBD point mutation in H505A maintains the ability to generate an inhibitory antibody response equal to or better than the wild-type antigen.

### 3.5. Immunized Sera Protect from SARS-CoV-2 Infection

To verify that antibodies generated in mice against RBD mutants retained sufficient neutralizing capacity for a vaccination strategy, we evaluated the ability of H505A to induce a neutralizing response. Neutralization of infectious SARS-CoV-2 (Omicron sub-lineage B.1.1.529) virus, performed by plaque reduction assay, confirmed that sera from mice immunized with H505A neutralized the virus to a similar extent as sera from mice immunized with the wtRBD (Figure 2E).

Altogether, our findings confirm that the H505A RBD mutant displays reduced interaction with the ACE2 receptor, while still inducing a good neutralizing response when used in a vaccination strategy.

## 4. Discussion

This work demonstrates the technical feasibility of rationally engineering viral antigens to eliminate specific host receptor interactions while preserving immunogenicity. We successfully applied this approach to SARS-CoV-2; nonetheless, the methodology could potentially be extended to other pathogens where similar design considerations are relevant.

In SARS-CoV-2, as a proof of concept, we impaired the interaction between spike and ACE2 while maintaining vaccine immunogenicity. This approach provides a valuable research tool for investigating the potential role of spike-ACE2 interactions in vaccination outcomes and serves as a foundation for rational vaccine design strategies.

We generated single-point RBD mutants with vastly diminished interaction with ACE2 but preserved, and in the case of H505A, enhanced ability to elicit a protective antibody response in vaccinated mice. These findings demonstrate that essential vaccine functions can be maintained while limiting specific antigen–receptor interactions.

We employed computer-assisted rational protein engineering to design mutants, prioritizing single-point mutations to minimize conformational changes and ensure the generation of antibodies that effectively recognize the virus.

In vivo mouse immunization revealed that these mutants elicited a robust, neutralizing immune response against infectious virus. Extensive literature confirms a direct correlation between serum neutralization, as tested here, and protection from infection [32,33]. Notably, the H505A mutant induced the strongest neutralizing response against the tested SARS-CoV-2 Omicron sub-lineage B.1.1.529, exceeding the response of the wtRBD.

## 5. Conclusions

This work establishes a clear proof of concept for a rational antigen engineering strategy, demonstrating that critical vaccine immunogenicity can be successfully decoupled from host–receptor interactions. Through precise, single-point mutations, we have shown that viral antigens can be modified to impair receptor binding without compromising their capacity to elicit robust, neutralizing antibody responses in vivo.

This rational design approach may be relevant to other pathogens that utilize entry receptors with critical physiological roles, offering a framework for investigating antigen–receptor interactions in vaccine development. However, the clinical significance of eliminating the adverse effects warrants validation through appropriate safety and efficacy studies.

## Data Availability

Data is contained within the article or the Supplementary Materials.

## Supplementary Materials

The following supporting information can be downloaded at: https://www.mdpi.com/article/10.3390/vaccines14070568/s1, Table S1: Summary of expression yields for recombinant wtRBD and selected mutants; Figure S1: Sera from mice immunized with wtRBD or specific RBD mutants were analyzed via ELISA for binding affinity to recombinant wtRBD. "M" corresponds to the individual mouse number; Figure S2: Competitive ELISA showing the ability of sera from mice immunized with RBD mutants to block the interaction between wtRBD and the ACE2 receptor. "M" corresponds to the individual mouse number.
