# Supplementary material for: Rationally Modified SARS-CoV-2 Spike Protein Impairs ACE2 Binding While Preserving Immunogenicity in Mice
Source: Vaccines (Basel). 2026 Jun 27;14(7):568. doi: 10.3390/vaccines14070568 (PMC13418380; doi:10.3390/vaccines14070568)
Supplement: Supplementary file 1 [file vaccines-14-00568-s001.zip › vaccines-4382534-supplementary.pdf]

Recombinant protein	Production yield (~mg of protein per L of culture)
RBD WT	**23**
RBD Y453A	**2**
RBD Y489A	**3**
RBD R493A	**4**
RBD R498A	**6**
RBD Y501A	**27**
RBD H505A	**24**

Table S1. Summary of expression yields for recombinant wtRBD and selected mutants.

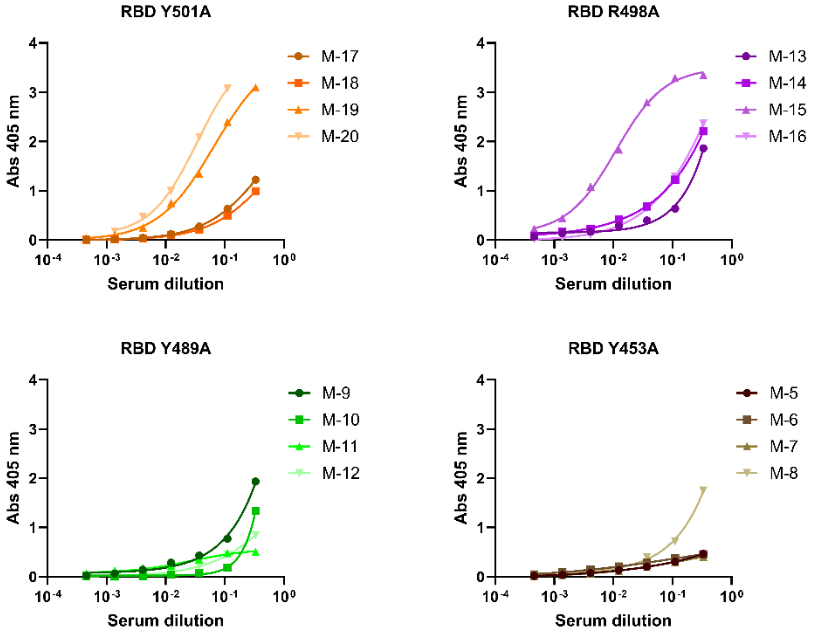

Fig. S1. Sera from mice immunized with wtRBD or specific RBD mutants were analyzed via ELISA for binding affinity to recombinant wtRBD. "M" corresponds to the individual mouse number.

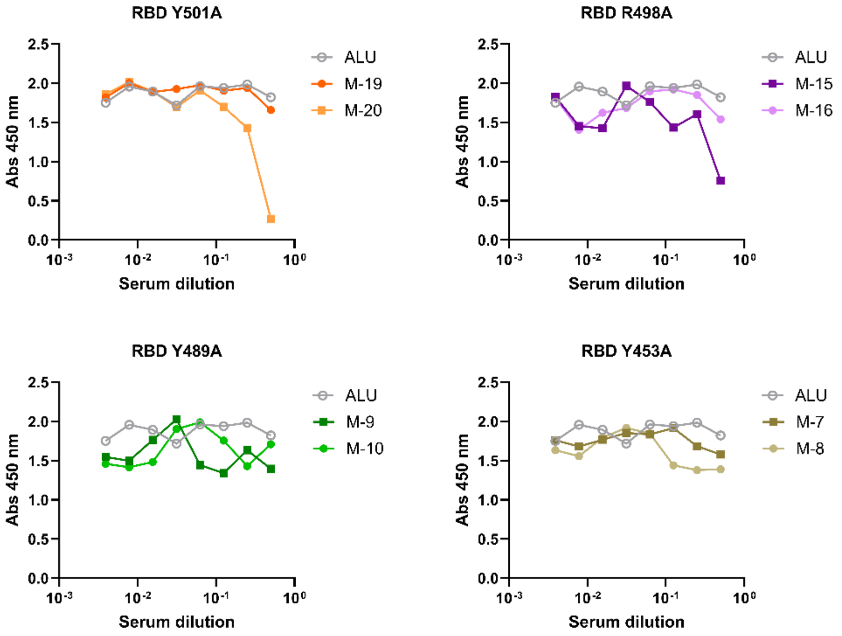

Fig. S2. Competitive ELISA showing the ability of sera from mice immunized with RBD mutants to block the interaction between wtRBD and the ACE2 receptor. "M" corresponds to the individual mouse number.
